# Check and Report Ebola (CARE) Hotline: The User Perspective of an Innovative Tool for Postarrival Monitoring of Ebola in the United States

**DOI:** 10.2196/publichealth.7817

**Published:** 2017-11-14

**Authors:** Ilana Olin McCarthy, Abbey E Wojno, Heather A Joseph, Scott Teesdale

**Affiliations:** ^1^ Division of Global Migration and Quarantine Centers for Disease Control and Prevention Atlanta, GA United States; ^2^ Innovative Support to Emergencies, Diseases, and Disasters Sunnyvale, CA United States

**Keywords:** Ebola, postarrival monitoring, interactive voice recognition

## Abstract

**Background:**

The response to the 2014-2016 Ebola epidemic included an unprecedented effort from federal, state, and local public health authorities to monitor the health of travelers entering the United States from countries with Ebola outbreaks. The Check and Report Ebola (CARE) Hotline, a novel approach to monitoring, was designed to enable travelers to report their health status daily to an interactive voice recognition (IVR) system. The system was tested with 70 Centers for Disease Control and Prevention (CDC) federal employees returning from deployments in outbreak countries.

**Objective:**

The objective of this study was to describe the development of the CARE Hotline as a tool for postarrival monitoring and examine the usage characteristics and user experience of the tool during a public health emergency.

**Methods:**

Data were obtained from two sources. First, the CARE Hotline system produced a call log which summarized the usage characteristics of all 70 users’ daily health reports. Second, we surveyed federal employees (n=70) who used the CARE Hotline to engage in monitoring. A total of 21 (21/70, 30%) respondents were included in the survey analytic sample.

**Results:**

While the CARE Hotline was used for monitoring, 70 users completed a total of 1313 calls. We found that 94.06% (1235/1313) of calls were successful, and the average call time significantly decreased from the beginning of the monitoring period to the end by 32 seconds (*Z* score=−6.52, *P*<.001). CARE Hotline call log data were confirmed by user feedback; survey results indicated that users became more familiar with the system and found the system easier to use, from the beginning to the end of their monitoring period. The majority of the users were highly satisfied (90%, 19/21) with the system, indicating ease of use and convenience as primary reasons, and would recommend it for future monitoring efforts (90%, 19/21).

**Conclusions:**

The CARE Hotline garnered high user satisfaction, required minimal reporting time from users, and was an easily learned tool for monitoring. This phone-based technology can be modified for future public health emergencies.

## Introduction

The 2014-2016 Ebola epidemic in West Africa was an unprecedented public health emergency afflicting more than 28,000 people and claiming more than 11,000 lives [1]. The Centers for Disease Control and Prevention (CDC) extended international efforts to control and prevent disease spread in West Africa and implemented domestic measures to prevent the introduction and transmission of Ebola in the United States [2,3]. In late October 2014, CDC recommended postarrival monitoring of all travelers arriving to the United States from countries with Ebola outbreaks and called on state and local public health authorities to monitor travelers for signs and symptoms of Ebola at least once daily for 21 days following each traveler’s last possible exposure [4]. The purpose of postarrival monitoring was to ensure that people with epidemiologic risk factors who became ill were identified as soon as possible after symptom onset to be quickly isolated, evaluated, and treated, if necessary [5].

In response to CDC’s recommendation [6], state and local public health authorities quickly initiated programs to monitor all potentially exposed travelers arriving within their jurisdictions. To aid state and local public health authorities in postarrival monitoring and as part of a larger effort to engage with and educate travelers at a number of the US ports of entry, CDC provided all travelers arriving to the United States from countries with Ebola outbreaks with a prepaid cell phone with short message service (SMS) text capabilities [2,3]. The purpose of this was two-fold; first, to provide travelers with a means to connect with public health authorities in the event they did not have a cell phone and, second, to ensure that public health authorities had phone numbers for travelers in their jurisdictions.

State and local public health authorities utilized various reporting mechanisms, including in-person health checks, centralized call centers, SMS text reporting of symptoms, mobile apps, and Web-based reporting systems to collect the daily health reports [7-10]. Although the systems differed in approach, a common theme was the need for supplementary resources beyond routine funding, personnel, and technology for the rapid and effective establishment of monitoring systems [3,11,12].

This report focuses on an innovative monitoring system, the Check and Report Ebola (CARE) Hotline, which allowed travelers to report their daily health status to an interactive voice recognition (IVR) system. The CARE Hotline was developed through a collaboration between CDC and Innovative Support to Emergencies, Diseases, and Disasters (InSTEDD, a nonprofit technology organization based in Sunnyvale, California), with support from Skoll Global Threats Fund. The goal of the partnership was to provide state and local public health authorities with an efficient and effective tool that could be used to conduct monitoring. More specifically, the intended result was to build and deploy a system that allowed travelers to fulfill monitoring requirements and provided public health authorities with timely and accurate data that met the changing needs of the outbreak response. InSTEDD contributed their open-source technology, Verboice (IVR software) and mBuilder (SMS text software), to design the CARE Hotline interface.

The aim of this evaluation was to describe the usage characteristics and user experience of the CARE Hotline as a tool for postarrival monitoring. Similar telephonic monitoring systems have been used in other communicable and noncommunicable disease monitoring and surveillance activities [13-21]. High user satisfaction, usability, and adherence have been found in IVR, Web-based, and SMS text monitoring systems for chronic health conditions [18,21]. Low usability and adherence has been found in infectious disease medication monitoring in a resource-limited setting [14]. However, on the basis of our review of the literature, the usage characteristics and user experience of an IVR to conduct required monitoring in an emergency response are still unknown. To the best of our knowledge, this is the first report describing and evaluating a combined IVR and SMS system for postarrival monitoring for Ebola.

## Methods

### IVR Interface and SMS Feature

The CARE Hotline included a simple IVR interface with three prompts and an SMS feature that pushed text reminders to elicit travelers’ participation. Users were provided with a phone number for the CARE Hotline and were instructed to call daily for monitoring. Users who failed to call into the system by 3:00 PM each day received an SMS reminder prompting report of health status. The CARE Hotline was developed with standard scripts, including an initial registration call script that was intended to provide users with information about the hotline, monitoring of symptoms, and to introduce them to the interface as well as the daily health report script. CDC and InSTEDD designed the health report script with the following three yes/no answer-format questions related to key monitoring requirements:

1. Have you taken your temperature today?
2. Is your temperature at or above 99.5º Fahrenheit?
3. Do you have other symptoms such as severe headache, muscle pain, weakness, diarrhea, vomiting, stomach pain, or unexplained bleeding or bruising?

Those who reported fever or symptoms were immediately transferred to a live person. Additionally, users could request to interact in real time with a person whether or not they exhibited symptoms. Lastly, scripts were created in English and French, the two most common languages of travelers arriving from outbreak countries.

CDC deployed a live version of the CARE Hotline on November 26, 2014, approximately 1 month after US public health authorities initiated their monitoring programs. This live version of the CARE Hotline was tested among CDC federal employees returning from deployments in outbreak countries. While the CARE Hotline project team managed the system, CDC’s Occupational Health Clinic oversaw the monitoring of employees and and followed up with noncompliant users—users who failed to report their health status in a 24-hour period. Starting November 26, all returning CDC employees based in Atlanta were enrolled in the system and were instructed to call the CARE Hotline to submit their daily health reports. Health reports were collected through January 18, 2015. During that time, 70 employees used the system for monitoring. In addition to using the CARE Hotline to engage in monitoring, CDC employees were simultaneously required to report daily to their state or local public health authority.

### Data Collection

While the CARE Hotline was being used as a monitoring tool by CDC employees, it generated a call log that summarized usage characteristics, including call length, frequency of calls, responses to the three prompting questions, and referrals to a live person. These data were used for the call log analyses.

CDC developed a Web-based survey using SurveyMonkey (SuveyMonkey Inc, San Mateo, California). The survey included 25 open- and closed-ended questions that assessed perceptions of the CARE Hotline, including ease of use, familiarity, confidence, and satisfaction. The closed-ended questions were categorical, dichotomous, or based on a 6-point Likert scale. The survey was pilot-tested using cognitive interviewing, and survey questions were revised based on feedback from matched CDC volunteers (CDC employees who returned from deployments in outbreak countries and fulfilled postarrival monitoring requirements using a different system than the CARE Hotline). The survey was distributed approximately 1 year after employees used the CARE Hotline. To reduce recall bias and encourage familiarity, survey respondents were prompted to call the CARE Hotline before completing the survey.

Participants were asked to provide their name when completing the survey to pair their survey responses to their call log data. The data collection was therefore confidential but not anonymous. The evaluation was reviewed and approved as a public health response program evaluation activity and not as research involving human subjects.

### Sample

During CDC’s live test of the CARE Hotline from November 26, 2014, through January 18, 2015, a total of 70 employees were enrolled in the system for monitoring. Call log data from all users (n=70) were included in the analyses of the call metrics. All employees who used the system (n=70) were invited by email to complete the survey voluntarily. A total of 26 (37%) of the 70 eligible employees completed the survey, but 5 respondent surveys were excluded from the final analyses because of incomplete survey response, leaving an analytic sample of 21 (30%).

### Analysis

#### Call Log Metrics

Call log data for all users (n=70) were analyzed in Microsoft Excel and SPSS version 21. Standard descriptive statistics were used to summarize all call log data (n=1313 calls). To compare average call lengths derived from the first three calls, after registration, and the last three calls, the nonparametric Wilcoxon signed-rank test was used since the data were not normally distributed. First, we used the Tukey method to identify extreme outliers in this set of call logs [22]. Extreme outliers are defined as those that are 3 times the interquartile range (IQR=59 seconds) for the upper (3rd quartile; 1 minute and 24 seconds) and lower (1st quartile; 25 seconds) quartiles. For all call log data (n=1313 calls), the upper bounds were determined to be 4 minutes and 21 seconds (3rd quartile + [3×IQR]). Therefore, for this mean comparison analysis, 12 calls lasting longer than 4 minutes and 21 seconds were removed from the call log data.

#### Survey Feedback

Quantitative survey data (n=21) were imported into Microsoft Excel for analysis. Standard descriptive statistics were used to summarize these data. Qualitative data were imported into Microsoft Excel, and an applied thematic approach was used; that is, raw data from open-ended fields were reviewed and common themes were coded by a single analyst and then enumerated and summarized.

## Results

### Call Log Metrics

Call data were pulled from the CARE Hotline call log. On average, the 70 users reported to the system for 16 days of monitoring and made 19 calls per user for a total of 1313 calls to the CARE Hotline. Of the total, 94.06% (1235/1313) of those calls were successful where users completed the IVR prompts and (1) reported that they were healthy (n=1208, 92.00% of all calls) or (2) reported they had fever and/or symptoms *and* were connected to a live representative (n=18, 1% of all calls) or (3) requested to be *and* were connected to a live representative (n=9, 1% of all calls). Of the total, 4.72% (62/1313) of calls were coded as unsuccessful in that users (1) did not complete the three IVR prompts (46/1313, 3.50% of all calls) or (2) were not connected to a live representative when reporting fever or symptoms (16/1313, 1.22% of all calls). By reviewing unsuccessful call logs, we noted two repeat behaviors related to the 62 unsuccessful calls: 74% (46/62) of users ended existing calls and called back within 2 hours to rereport healthy symptoms, and 26% (16/62) of users ended calls during the referral process to a live representative. Lastly, due to a technical error in late December 2014, time data for 1.22% (16/1313) of all calls were collected, but IVR prompt responses were not recorded and could not be retrieved. Therefore, we are unable to categorize those calls as successful or unsuccessful.

As expected, registration calls (n=92, users could register multiple phone numbers), which contained additional script, lasted longer than subsequent calls for an average of 2 minutes and 54 seconds. Therefore, the registration call was excluded from the Wilcoxon signed-rank test. A total of 67 users had at least 6 call records and were included in this analysis. This test required that users have a minimum of 6 calls to be included in the analysis; the average of the first three calls was compared with the average of the last three. For this Wilcoxon signed-rank test, we found that call time decreased for users (n=67, 402 calls included in this analysis) from the beginning to the end. The average call time for the first three calls was 1 minute and 14 seconds but was reduced to 42 seconds for the last three calls (*Z* score=−6.52, *P*<.001).

The majority of calls, excluding the initial registration call (747/1221, 61.18%), were made before 3:00 PM and, thus, there were only a total of 474 SMS reminders. Of the total, 24.1% (114/474) of SMS reminders were successful at nudging users to report to the system within 2 hours. The remaining 75.9% (360/474) did not increase user compliance within 2 hours. Administrators of the CARE Hotline in CDC’s Occupational Health Clinic were alerted to follow up 95 times during the live test. Alerts to follow-up occurred if users failed to submit their health report within a 24-hour period.

### Survey Feedback

As users progressed in their monitoring period, they reported becoming more familiar with the system and their confidence in reporting accurately to the system increased. Overall, 19 of 21 respondents (90%) reported that the system was “very easy” (n=15) or “easy” (n=4) to use for monitoring. 

**Table 1:** Key themes and illustrative responses.

Variable and theme			Response
**Ease of use**			
	Simple instructions	The instructions were straightforward and answering the questions was easy	
		Simple instructions, quick process, minimal fuss; automated and rapid	
	Easy system	Simple, easy task	
		Easy due to use of technology in performing day-to-day tasks	
		The process was easy to follow	
	Straightforward system	It was straightforward, and it worked	
		Very straightforward and did not require much time investment to file accurate and timely reports	
**Overall satisfaction**			
	Easy, convenient, and unobtrusive	Easy to use and nonintrusive, so I could fit in my schedule without disruption	
		Simple, easy to do, unobtrusive	
		Given the required frequency of monitoring, the system could not have been easier or [more] convenient to use	
		The overall process was not invasive and allowed me to monitor health while continuing with day-to-day activities	
		Could get used to prompts and pattern, use it quickly and when it was convenient in my schedule	
	Effective solution	Very satisfied—I cannot think of changing anything to improve the process, technology, or system	
		The hotline was easy to use and an effective way to monitor returning travelers	
		I thought it was a great solution to the active monitoring requirement	
**Recommended improvements**			
	Recommendations (many respondents indicated no recommendations)	Develop a phone app	
		Text option; ability to skip the introductory info[rmation] after, say, day 3	
		I think something a bit simpler would be better, especially a text with instructions every day, to which someone could respond with one letter or word	
		Texting may be even better than calling in and dealing with the prompts	

More than three-quarters (17/21, 81%) noted they became more familiar with the CARE Hotline and the three prompts during their monitoring period; the remaining four could not remember. The majority (86%) of respondents reported feeling “very confident” (11/21) or “confident” (7/21) in submitting their first health report to the system; the remaining 3 (14%) felt “somewhat confident.” After using the system, confidence increased; all reported feeling “very confident” (17/21, 81%) or “confident” (4/21, 19%) in submitting their last report to the system.

Of the 21 respondents, 11 indicated receiving an SMS reminder. When asked about their experience with the SMS feature, all 11 participants indicated that this feature was “very helpful” or “helpful” in prompting them to submit a health report. Of the 11 respondents, 7 (64%) reported that without this feature, they would have missed a day of monitoring.

When asked to indicate their overall level of satisfaction with the CARE Hotline, all responded favorably; 90% (19/21) reported being either “very satisfied” (n=11) or “satisfied” (n=8), and 10% (2/21) reported being “somewhat satisfied.” Almost all (19/21, 90%) would recommend the system for monitoring. When asked to explain why they were satisfied, ease and simplicity were the most common themes. Users liked being able to call at their convenience, as well as the ability to move quickly through the prompts. The most commonly offered suggestion for improvement was to allow users to submit reports entirely via texting or a mobile app. Table 1 highlights qualitative responses that respondents provided for their ratings for the following variables: ease of use, overall satisfaction, and recommendations for improvement.

## Discussion

### Principal Findings

The CARE Hotline was quickly built, deployed, and tested by CDC employees as a monitoring system during the Ebola emergency response. Our evaluation of the live test demonstrated that the vast majority of CDC users were able to successfully engage in monitoring using the CARE Hotline. Additionally, they were adequately confident in their ability to use the system initially, becoming more confident over time. Users identified ease of use, convenience, and the unobtrusive nature of the system as main factors in their overall satisfaction. Users’ confidence with the technology and the ease of using the tool made the quick user adoption of the CARE Hotline feasible.

The tech components of the system required minimal funds; the hotline phone number, IVR, and SMS cost less than US $42 total to operate. Cost data are based on the establishment and use of the hotline number (2 lines for production and 2 lines for staging, costing US $1 per month for a total of US $12 for 3 months), IVR minutes (US $0.0085 per minute), and the number of SMS texts (US $0.0075 per SMS) sent. These ongoing costs appear less than other IVR systems that report US $0.23 per minute and US $0.05 per SMS [14].

### Limitations

Although these findings are positive in terms of the user experience and the ability to quickly and successfully report to the CARE Hotline, we recognize the limitations in this sample. First, there was approximately a year lag time between engaging with the CARE Hotline and evaluating the user experience. However, attempts were made to reduce recall bias; users were provided with memory aids and guided through the CARE Hotline before completing the survey. Additionally, in the survey instructions, users were reminded that they were reporting their health status simultaneously to state or local public health authorities; they were prompted to only consider their experiences with the CARE Hotline when answering survey questions. Second, the final survey respondent analytic sample contained 30% of the CARE Hotline users. Although this is small, we believe that this is a fair response rate for survey data and that the responses obtained provide important insight into the user experience. Lastly, we recognize that CDC employees may differ from the general public and, therefore, recommend future testing among a more diverse audience to help confirm these preliminary findings of high acceptability and feasibility. Even though these results are preliminary and there are limitations, this system should be considered as a resource-saving alternative to human-driven monitoring systems such as call centers for future monitoring efforts that may have inadequate financial and human resources.

### Considerations for Future Application

Beyond these results, several additional insights may be helpful when considering the use of a similar system in an emergency response. During the IVR development phase, CDC and InSTEDD followed an agile approach by managing the project iteratively and incrementally, with frequent conversations and short feedback loops. This encouraged stakeholder engagement, helped to confirm design decisions, and ensured high quality. CDC and InSTEDD developed and deployed early versions of the CARE Hotline within a condensed time frame. The CARE Hotline moved from an idea to a tested functional system within weeks.

The use of flexible, easy, and free open-source tools kept the development and launch costs low. The team relied on InSTEDD’s mBuilder and Verboice tools to integrate voice and SMS capabilities. These tools can be used off the shelf and allowed for customization to meet specific and evolving needs. Such free and open-source technologies can be used individually or as building blocks for larger solutions tailored to support future public health emergencies. Additionally, support from Skoll Global Threats Fund served as a catalyst for this effort and demonstrates the role philanthropy and established partner networks can play in helping even the largest institutions stay nimble and explore innovative techniques.

When designing an IVR system to support an emergency response, basic user requirements should be considered. IVRs require access to phone service. Landlines and mobile phones can be used to submit reports to the system, although mobile phones with text capabilities would be required of IVR systems that depend on SMS components for engaging or nudging users. IVRs require that users can easily access the system, input reports, and reply to text messages for systems that include SMS functionality.

When determining whether to deploy an IVR, audience demographics, including primary languages, should be considered. Nonetheless, IVRs can easily support various languages and dialects. When resources permit, call scripts can be translated and recorded by voice talent. Quality assurance measures ensure that the prompts are understood by native speakers and reflect differences between dialects.

Ultimately, the CARE Hotline was not adopted following the CDC live test for a number of reasons. State and local public health authorities had, by that time, implemented their own monitoring systems. In addition, monitoring strategies varied between states and even among local jurisdictions within a state as authorities tailored their systems to the local need. This complicated the deployment of a standardized and centralized monitoring system at the state or local level. There were also concerns about implementing a newly developed automated, technology-based system for Ebola postarrival monitoring before thoroughly evaluating its effectiveness and reliability.

Should states need to perform monitoring following communicable disease exposures in a future public health emergency, an IVR such as the CARE Hotline could be implemented on a state or federal level. Future administrators might consider additional mobile phone functionality such as graphic and audio file sharing. The system could be adapted for other public health response efforts, such as monitoring during contact investigations, for surveillance purposes, or for health education or promotion purposes [15,16]. Such a system could be programmed to share health information, including, but not limited to, emergency updates, instructions, reminders for medication, or prevention messaging for infectious disease outbreaks. As outlined by Patrick et al [16], evidence is emerging that supports the effectiveness of mobile phones for diagnosis, management, and treatment of disease, along with health promotion and prevention messaging. A high successful usage rate, positive user experience, and system adaptability are key for the potential deployment of similar systems in routine and future outbreak-related monitoring and surveillance efforts. The IVR described here might have applications far beyond postarrival monitoring for Ebola.

## Abbreviations

CARE: Check and Report Ebola
CDC: Centers for Disease Control and Prevention
InSTEDD: Innovative Support to Emergencies, Diseases, and Disasters
IVR: interactive voice recognition
IQR: interquartile range
SMS: short message service
